# Monotonic and Cyclic Bond Behavior of Deformed CFRP Bars in High Strength Concrete

**DOI:** 10.3390/polym8060211

**Published:** 2016-05-31

**Authors:** T. Tibet Akbas, Oguz C. Celik, Cem Yalcin, Alper Ilki

**Affiliations:** 1 Institute of Science and Technologie, Istanbul Technical University, 34469 Istanbul, Turkey; 2 Department of Architecture, Istanbul Technical University, 34437 Istanbul, Turkey; celikoguz@itu.edu.tr; 3 Department of Civil Engineering, Bogazici University, 34342 Istanbul, Turkey; yalcince@boun.edu.tr; 4 Department of Civil Engineering, Istanbul Technical University, 34469 Istanbul, Turkey; ailki@itu.edu.tr

**Keywords:** bond strength, CFRP, cyclic, pullout, high strength concrete

## Abstract

Composite reinforcing bars (rebars) that are used in concrete members with high performance (strength and durability) properties could have beneficial effects on the behavior of these members. This is especially vital when a building is constructed in an aggressive environment, for instance a corrosive environment. Although tension capacity/weight (or volume) ratios in composite rebars (carbon fiber reinforced polymer (CFRP), glass fiber reinforced polymer (GFRP), *etc.*) are very high when compared to steel rebars, major weaknesses in concrete members reinforced with these composite rebars may be the potential consequences of relatively poor bonding capacity. This may even be more crucial when the member is subjected to cyclic loading. Although monotonic bond tests are available in the literature, only limited experimental studies exist on bond characteristics under cyclic loading conditions. In order to fill this gap and propose preliminary design recommendations, 10 specimens of 10-mm-diameter ribbed CFRP rebars embedded in specially designed high strength concrete (*f*’_c_ = 70 MPa) blocks were subjected to monotonic and cyclic pullout tests. The experimental results showed that cyclically loaded CFRP rebars had less bond strength than those companion specimens loaded monotonically.

## 1. Introduction

Reinforcements made of carbon fiber are widely used as retrofitting materials to increase the capacity of existing buildings [1]. Recent studies have shown the usage of carbon fiber reinforcing bars in concrete members effectively as an alternative material to steel rebars. Thus, fiber reinforced polymer (FRP) reinforced concrete members such as beams, columns and slabs could be designed and constructed. Carbon rebars are mostly preferred in harsh environments due to their chemical resistivity, thermal conductivity, electrical conductivity, dimensional stability and durability [2,3]. High strength and light weight characteristics are other remarkable features of these rebars. Some construction applications with carbon rebars in marine structures, bridge concrete slabs and concrete pavements have already proved their efficiency and suitability under corrosive exposure conditions. Research in this field has also gained some momentum in terms of assessing their potential in the future.

Recently conducted experimental studies by various researchers showed similar behavioral properties of carbon rebars in concrete sections when compared to steel rebars. These studies also revealed that required embedment lengths of these rebars should be taken into consideration correctly and carefully. Unlike steel rebars, less information is available in the literature on the bond behavior of carbon rebars in normal and high strength concrete members, particularly for cyclic loading conditions.

In the past decades researchers have carried out numerous experimental studies and proposed bond models to describe the behavior of steel rebars in terms of their embedment and anchorage lengths. Alsiwat and Saatcioglu [4] formulated a bond-slip model using existing test results of distribution and transfer of forces between steel reinforcement and concrete. Other researchers worked on the impact of reinforced concrete (RC), detailing the bond-slip relationship where they evaluated the bond behavior for different conditions of concrete cover and confinement [5]. According to detailed experimental studies conducted using direct tension pullout tests, it has been observed that the force distribution was not uniform along the rebar embedment length [6].

Similar to steel rebars, composite rebars also do not uniformly transfer forces to concrete [7]. Steel rebar surface properties are standardized as a result of past experimental studies. However, composite rebars are produced with various base materials such as carbon, glass or aramid, and may have different surface conditions such as ribbed or roughened. Therefore, there is still a lack of sufficient experimental background and standardization. It should also be noted that plain composite bars have only negligible bond resistance even in high strength concrete. American Concrete Institute ACI 440 [8] recommends basic formulations for the calculation of the required embedment length of composite rebars. Harajli and Abouniaj [9] have stated that these formulations are too conservative and not realistic. Other researchers’ studies also support this conclusion about the bond requirements of ACI 440 [10].

This experimental study mainly focuses on comparing the bond behavior of carbon fiber reinforced polymer (CFRP) rebars in a high strength concrete block under monotonic and cyclic loading conditions. In addition to the loading procedure, various embedment lengths were also taken as test parameters. Obtained results showed that the sequence of loading has a significant impact on the behavior of CFRP reinforced concrete members.

## 2. Specimen Preparation

All rebars had the same diameter (d) of 10 mm and were placed concentrically in the concrete blocks. According to the predetermined specimen sizes, 10 plywood molds have been manufactured with 350 mm × 350 mm × 300 mm dimensions. In this research, the concrete cover was kept constant and taken as two times the diameter of the rebar to conform to the ACI 440 [8] guideline. The concrete blocks were designed in a U-shaped plan configurationby using polystyrene foam elements with 150 mm × 150 mm × 300 mm dimensions) to provide the CFRP bars with the desired concrete cover. In order to provide full fixity of the concrete specimen at the base of the experimental setup, four steel pipes were placed at each corner of the specimen’s mold. The specimens were then fixed to the base via four threaded steel bars. In order to determine the variation of bond stress distribution along the CFRP rebar embedded inside the concrete block, five different embedment lengths were considered as multiples of the bar diameters (5 d, 10 d, 15 d, 20 d and 25 d). To obtain the intended embedment length, an unbonded surface is provided by means of wrapping the rebars with soft plastic pipes at two ends of the embedded parts. Although the prepared test setup was suitable for concentric and eccentric loading of the CFRP rebars, the specimens have been tested concentrically in this experimental work.

## 3. Materials

**Figure 1:**
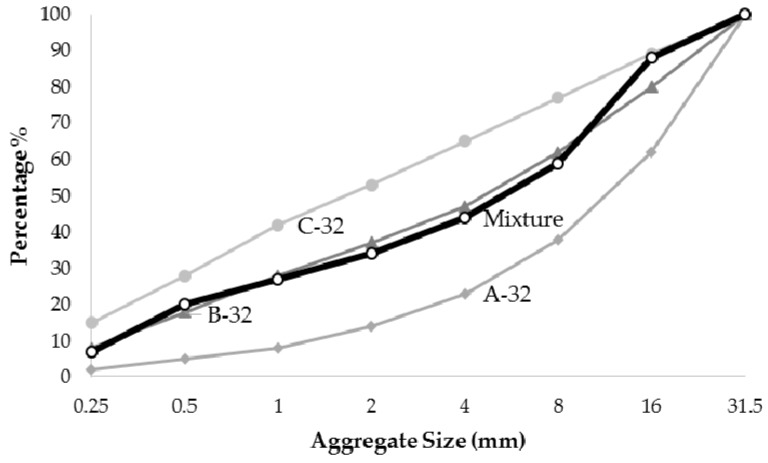
Mixture design.

Unconfined specimens were designed and constructed. As stated in the preceding section, the concrete cover was kept constant in order to observe the effect of 2 d cover spacing as per the ACI 440 guideline. It was thought that carbon rebars would be more appropriate to use in high strength concrete members [11]. As shown in Figure 1, an appropriate mix design was developed to target the C60 (cylindrical strength of 60 MPa) class of concrete as seen in Table 1. The standard cylinder specimens (150 mm × 300 mm) were tested under compression on the 28th day after pouring the concrete in place. Experimentally obtained average cylinder compressive strength was found to be 68.8 MPa.

**Figure 2:**
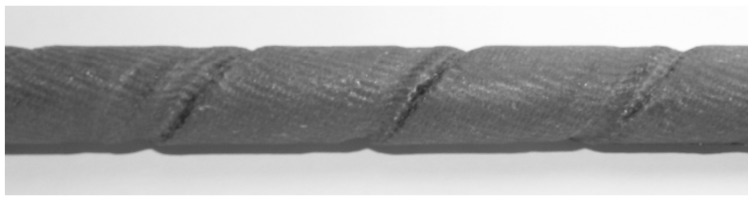
Rebar surface.

Sika CarboDur BC10 deformed rebars were selected in this research, as shown in Figure 2. The carbon rebars with *d* = 10 mm diameter and a 79 mm^2^ nominal cross-sectional area can resist 110 kN tension force under 1% elongation [12].

**Figure 3:**
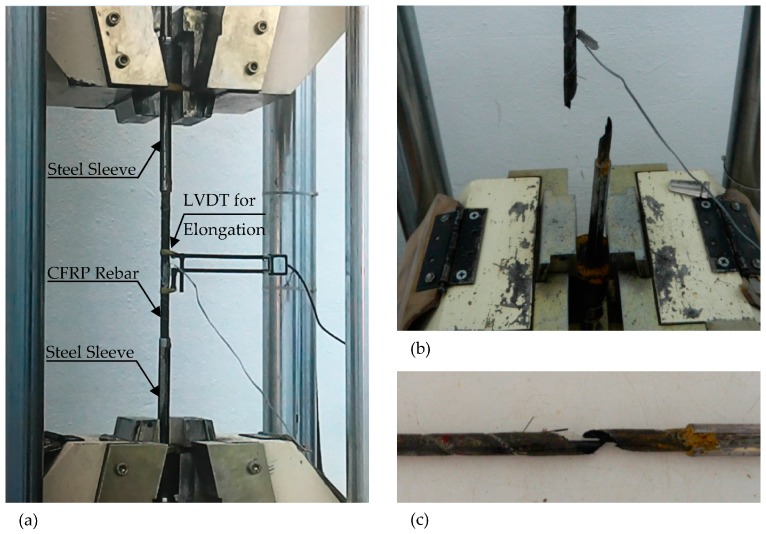
Rebar rupture test. (**a**) Test setup; (**b**) End of test; (**c**) Location of rupture.

Three CFRP rebar specimens were tested at the Materials Laboratory of Bogazici University (Figure 3). According to the test results, mechanical properties of CFRP rebars have been compared with the manufacturer’s suggested values as seen in Table 2. The measured average modulus of elasticity was 153,250 N/mm^2^. Measured rupture strengths for the specimens were 146.9, 104.4 and 126.6 kN, respectively. By using the average rupture value and also the experimentally obtained strength and modulus of elasticity values, it was found out that the fracture strain was slightly over 1.0%, which was different than the catalogue value.

## 4. Test Setup and Experimental Procedure

All specimens were tested at the Structures Laboratory of Bogazici University. The existing loading frame, which was used previously for steel rebar tests, was modified according to the requirements of this research. The test setup was equipped with special steel mounting parts to provide a reliable connection between the rebar and hydraulic jack (200 kN capacity with ± 200 mm of stroke). A special conical steel jaw attachment was designed to carry 250 kN of load without any slipping occurrences. The steel jaw assembly is the most crucial part of the experimental setup.

In order to obtain proper and accurate force distribution between the rebar and surrounding concrete, compatible strain gauges were chosen for the CFRP rebar surfaces and attached to various locations before concrete pouring.

Although CFRP rebars exhibit high tensile strengths in the longitudinal direction, they are weak in the transverse direction because of their orthotropic material properties. Without using any protecting cover for the rebar, the jaw would crush the rebar laterally, leading to erroneous results. For this reason, to prevent any material crushing, the tested rebars were surrounded with a 250-mm-long steel pipe sleeve with a diameter slightly larger than the rebars. The spacing between the rebar and steel sleeve was filled with a high strength epoxy having more than 3 MPa of bonding capacity to the steel.

**Figure 4:**
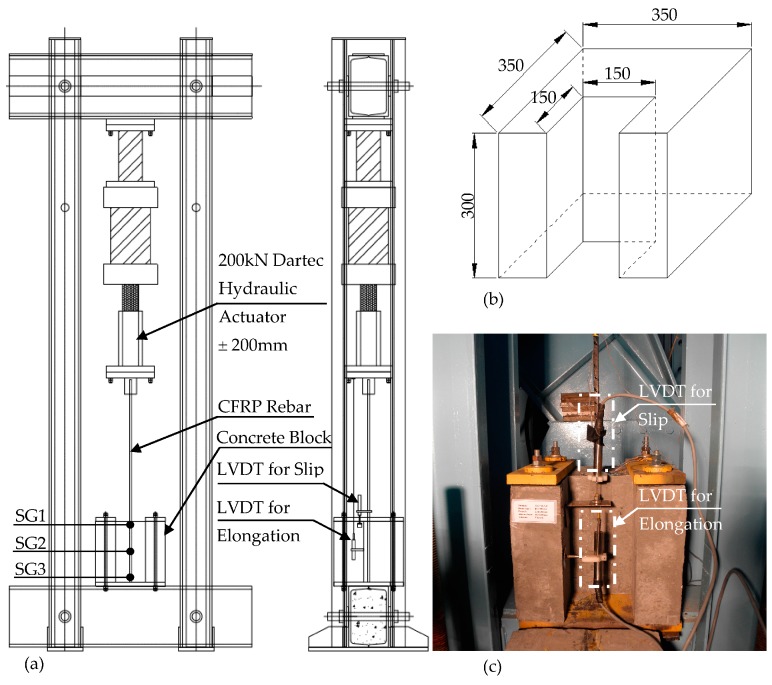
Test Setup. (**a**) Loading frame; (**b**) Concrete block dimensions; (**c**) LVDT location.

The schematic arrangement of the test setup is shown in Figure 4. A hydraulic jack was suspended from the top of the closed loading frame in the downward position with the conical jaw attached at the head of the actuator. The tip of the CFRP rebar was attached to the jaw. Specimens were seated on a steel plate having a thickness of 25 mm, which was welded to a concrete-filled steel foundation beam. The concrete block was then fixed with four threaded rods at the edges. There were two steel plates with a 25 mm thickness connecting two of these rods together at the top of the specimen, providing a full tension area between these steel plates.

As opposed to other proposed test setups (e.g., ASTM D7913) that produce compression on the concrete surface, the test setup developed in this work allows us to measure the bond capacity of CFRP rebars with the pure tension area in a concentric or eccentric loading configuration. Also, various embedment lengths from 5 d to 30 d with different concrete covers can be tested. Note that many test setups allow a 5 d embedment length. It is believed that the real behavior of the bond mechanism can be better achieved with the testing method developed in this work.

The specimens were tested monotonically and cyclically. In cyclic tests, only specimens with 25 d, 20 d and 15 d embedment depths were tested using the force-controlled test method with 5 kN of force increments and three repetitions. Displacement-controlled cyclic testing was performed for other specimens. Displacements were increased by 0.01 mm at a time.

## 5. Instrumentation

One Linear Variable Differential Transformer (LVDT) (100 mm) was connected to the rebar with a manacle to measure the relative slip (Figure 4c). Another LVDT was connected on the concrete block surface and also to the hydraulic jack with a wire.

In order to monitor the strain distribution inside the concrete block on rebar surfaces, three BFLA-5 type strain gauges (SG1, SG2 and SG3) were attached on the rebar. Strain gauge measurements were taken at the beginning of the bonded area (SG1), in the middle of the bonded area (SG2) and at the end of the bonded area (SG3) (Figure 4a).

## 6. Expected Failure Modes

**Figure 5:**
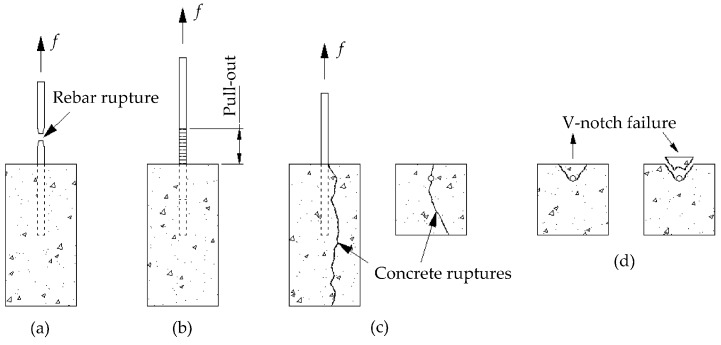
Expected failure modes. (**a**) Rebar rupture side view; (**b**) Pullout side view; (**c**) Splitting failure (shear type) side and top views; (**d**) Splitting failure (V-notch type) top views.

Expected failure modes are explained in this section by comparing the experimental and expected failure modes. Figure 5a shows the failure mode regarding a rebar rupture. This happens when the bar is adequately embedded in the concrete block. On the other hand, a typical pullout failure is shown in Figure 5b, which corresponds to an inadequate embedment length.

If an adequate embedment length is provided in an unconfined concrete, the pullout force could exceed the tension capacity of the concrete and a shear failure would occur. Splitting failure occurs when the surrounding concrete cannot resist the circumferential (radial) tensile stresses. Splitting generally occurs on the surface of the member as flexural cracks start forming and the rebar is stressed and elongated towards failure with a continuous longitudinal crack along the rebar. Splitting cracks generally occur on the concrete surface (along the bonded length) and propagate longitudinally and transversely as the rebar is axially stressed.

In general, two types of splitting modes were expected prior to testing: concrete rupture and V-notch failures. Concrete rupture is most likely to occur when the force exceeds the tension capacity of the unconfined concrete specimen (Figure 5c). As shown in Figure 5d, the V-notch failure (spalling of the cover) occurs when the concrete cover is relatively small.

The pullout mechanism may be defined in three different failure modes: rib crushing, failure of the surface and concrete crushing. Rib crushing occurs in raised types of ribs such as in the steel rebar. If the load exceeds the capacity of the rib, it starts bending or yielding. The CFRP rebars used in this work have indented ribs. Therefore, rib crushing is not an expected failure mode in the tests. However, failure of the surface or concrete crushing may occur according to the ratio of the rib height to the rib gap.

**Figure 6:**
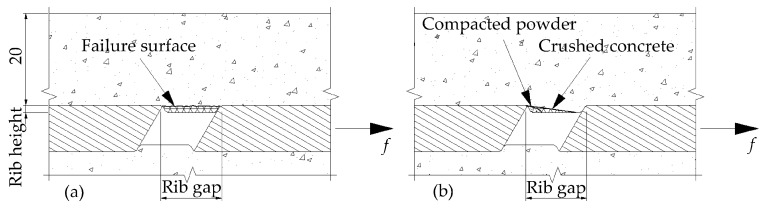
Failure mechanisms. (**a**) Failure of surface; (**b**) Concrete crushing.

When the ribs are high and spaced too closely, the shear stress may govern the behavior and the rebar would be pulled out (Figure 6a). As shown in Figure 6b, when the rib spacing is larger than approximately 10 times the rib’s height, the partly crushed concrete may form a wedge in front of the rib and failure is normally brought about with the splitting of the surrounding concrete [14].

## 7. Prediction of Strength According to ACI 440.R06

**Figure 7:**
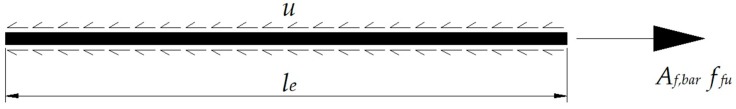
Transfer of force through bond.

Figure 7 shows the equilibrium condition of a CFRP rebar of length *l*_e_ embedded in a concrete block. The force in the rebar is resisted by an average bond stress *u* acting on the surface of the rebar. Equilibrium of forces can be written as follows;

(1) $$l_{e}\pi d_{b}u=A_{\text{f,bar}}f_{f}$$

where *A*_f,bar_ is the cross-section area of one rebar, *d*_b_ is the rebar diameter and *f*_f_ is the stress developed in the rebar at the end of the embedment length.

The development length equation for steel reinforcing bars found in ACI 318-08 [15] is based on the work done by Orangun [16]. A similar methodology was used by Wambeke and Shield for representing the bond performances of GFRP rebars [17]. According to the test results, they have derived an empirical bond formula. Based on their study, Equation (2) is proposed by ACI 440 [8] and recommended for bond capacity calculations of composite rebars.

(2) $$\frac{u}{0.083\sqrt{f_{c}{}^{\prime }}}=4.0+0.3\frac{C}{d_{b}}+100\frac{d_{b}}{l_{e}}$$

where *C* is the cover to the center of the rebar, *u* is the average bond stress acting on the surface of the GFRP rebar, *d_b_* is the diameter of the reinforcing bar, *f*_c_*’* is the specified compressive strength of the concrete and *l*_e_ is the embedded length of the reinforcing bar.

## 8. Experimental Results

Experimentally obtained test results are summarized in Table 3. This table includes specimen labels, embedment lengths, loading types, expected bond strengths and average bond stresses according to ACI 440 [8], measured bond strengths and average bond stresses according to the test results, and observed failure modes.

Expected bond capacities given in Table 3 are calculated from Equation (2) which is proposed by ACI 440 [8]. As seen from these results, measured bond stresses are generally higher than the calculated bond capacities according to ACI 440 [8]. For the embedment lengths of 25 d, 20 d and 15 d, measured bond strengths and stresses are almost twice the code-calculated values. However, similar bond capacities were obtained for 10 d and 5 d embedment length specimens.

Under monotonic testing, maximum axial loads of 28.3 and 86.4 kN were obtained for 5 d and 25 d specimens, respectively. As for cyclic (not reversed) loading, 16.2 and 70.0 kN load values were reached for 5 d and 25 d specimens, respectively. Developed bond stresses corresponding to these load values were 18.0 and 11.0 MPa for monotonic loading, and 10.3 and 8.9 MPa for cyclic loading. The experimental bond stresses could be significantly reduced when cyclic loads are applied. This results in reduced capacities for the specimens under cyclic loading. Reductions in short- and long-development-length specimens are 40% and 20%~25%, respectively. Experimental results showed that the attained minimum and maximum loads of 16.2 and 86.4 kN, respectively, were far below the maximum capacity (104.4 ~146.9 kN) of the deformed CFRP bars used.

**Figure 8:**
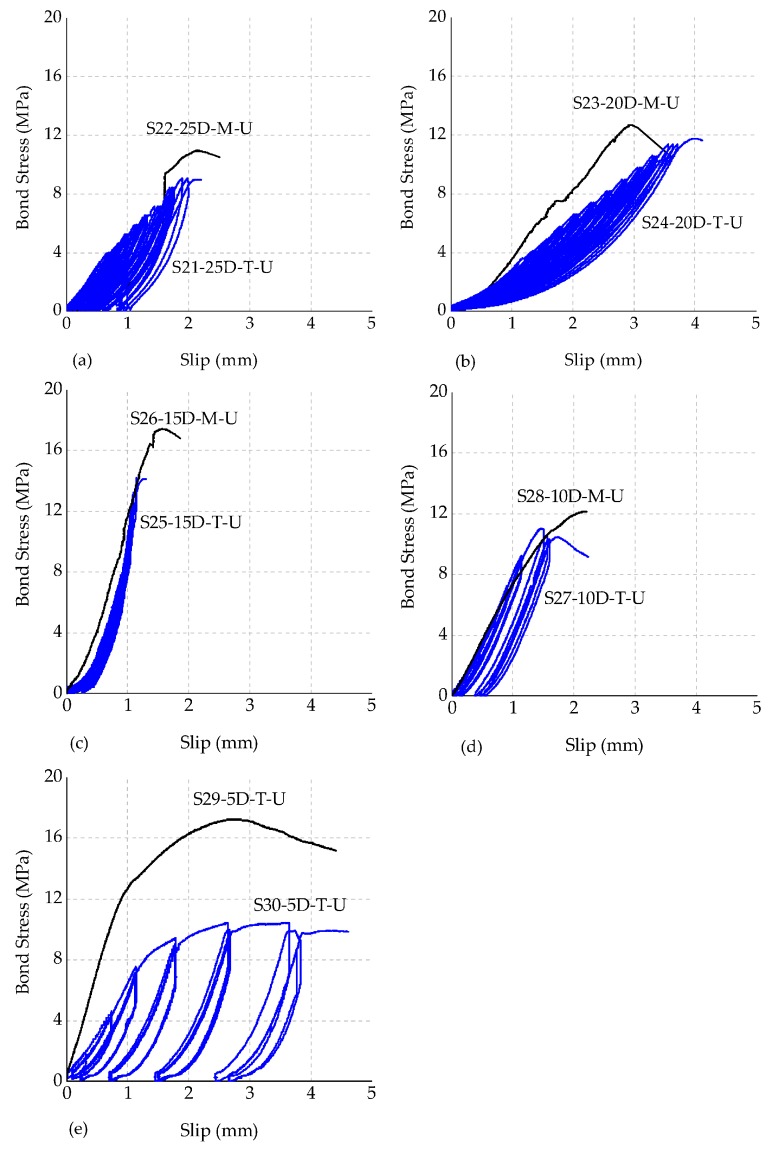
Experimental bond stress *versus* slip curves. (**a**) 25 d; (**b**) 20 d; (**c**) 15 d; (**d**) 10 d; (**e**) 5 d.

Bond stress (in MPa) *versus* slip (in mm) graphs are plotted and illustrated in Figure 8. It is recognized that the initial stiffness of the specimens changes depending on the loading procedure. In monotonically loaded specimens, measured bond stresses for shorter embedment lengths (for example for the 5 d specimen) are considerably greater than the stresses (max. 63.4% higher) that were obtained in specimens with longer embedment lengths.

Specimens S21, S22, S23 and S26 failed from the splitting mode while specimens S24, S25, S27, S28, S29 and S30 failed from the pullout failure mode. It was observed that splitting failure occurred under relatively high bond strengths, showing that the tensile strength of unconfined concrete controlled the behavior at this load level. For the splitting-type failures, loud sounds were heard, whereas for the pullout (or slip) failure no sound was heard. Since the concrete blocks were not confined, the splitting failures in some tests were quite brittle and caused complete wide cracks throughout the concrete block.

Figure 8 reveals that there is a significant behavioral variation between monotonic and cyclic test results. As a general observation from these figures, for specimens with 10 d or over, stiffness values are similar at early displacement levels. However, under cyclic loading, stiffness of the specimens tends to soften under larger displacement levels.

**Figure 9:**
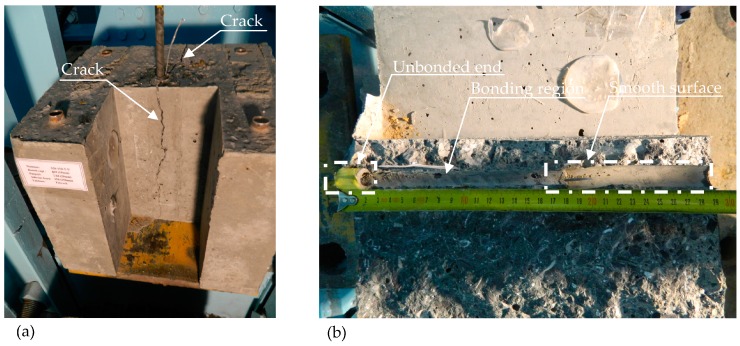
(**a**) Splitting failure; (**b**) Section of bonded and unbonded regions.

**Figure 10:**
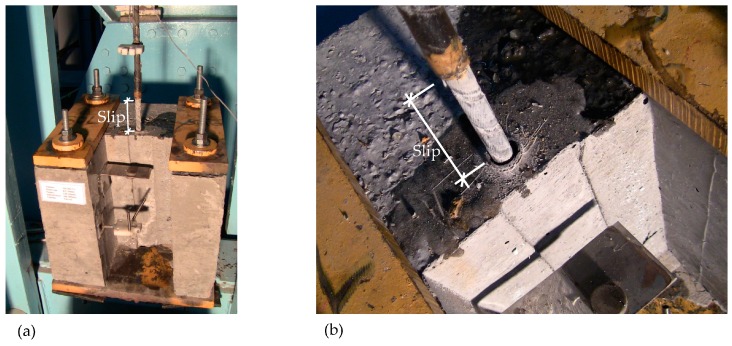
(**a**) Pullout failure; (**b**) No damage observed on concrete surface.

The general shapes of the behavioral curves obtained from this study were similar to the curves given in the existing literature. Typical failure modes for splitting and pullout failures are given in Figure 9 and Figure 10, respectively.

Achillides and Pilakoutas [18] tested CFRP rebars monotonically and calculated the average bond strengths between 9–12 MPa according to their experiments. Note that concrete cube compressive strength values of 40–50 MPa were used in their work. According to the obtained experimental results in the current study, the bond strength values changed between 10–18 MPa under monotonic loading and 9–14 MPa under cyclic loading. These results show that obtained monotonic loading capacities are remarkably higher than those obtained by the previous researchers [9,18]. This could be due to the better bond characteristics of the high strength concrete used in this study. Also, to the knowledge of the authors, although there are some experimental studies conducted with 30–50 MPa concrete strengths, limited experimental work exists for tests performed with high strength concrete (C60 and above).

## 9. Conclusions

Monotonic and cyclic experimental bond behavior of CFRP rebars in high strength (68.8 MPa) concrete was investigated. Various embedment lengths were studied. All (10) specimens had a constant concrete cover of 2 d. All monotonically-loaded specimens were loaded under a displacement-controlled testing procedure as well as the 5 d and 10 d embedded cyclic loading specimens. Other cyclic loaded specimens (25 d, 20 d and 15 d) were tested under the force-controlled testing procedure. The following conclusions could be drawn from this experimental work:

- In general, rebars with 5 d and 10 d embedment lengths failed due to pullout of the bar while rebars with 15 d, 20 d and 25 d embedment lengths failed due to splitting of the concrete. These failures were more brittle since the tension capacity of the unconfined concrete block was exceeded. Therefore, a thicker cover concrete (*i.e.*, >2 d) is more suitable for relatively deeper embedment lengths when high strength concrete is used.
- Stiffness of specimens varied significantly according to the selected loading protocols. Monotonically-loaded specimens had a higher stiffness when compared to the stiffness obtained for larger cycles of the cyclically-loaded specimens. However, generally, the stiffness values are similar (especially for embedment lengths of 10 d or longer) at early displacement levels for both cyclic and monotonic loading.
- Cyclic loading reduced the bond capacity of the specimens. Reductions in short- and long-development-length specimens are within the range of 40% and 20%~25%, respectively.
- For tested specimens, the bond strength values changed between 10~18 MPa under monotonic loading and 9~14 MPa under cyclic loading. These results showed that obtained monotonic loading capacities were considerably higher than those obtained by the previous research.
- Measured bond stresses are generally higher than the calculated bond capacities according to ACI 440 [8]. For the embedment lengths of 25 d, 20 d and 15 d, measured bond strengths and stresses are almost twice the code-calculated values. However, the calculated bond capacities were similar to obtained measurements for 10 d and 5 d embedment length specimens.

## Figures and Tables

**Table 1:** Mixture proportions used for the test series.

Mixture	Water/Cement	Water (kg/m^3^)	Cement (kg/m^3^)	Coarse Aggregate, 12–25 mm (kg/m^3^)	Coarse Aggregate, 6–12 mm (kg/m^3^)	Fine Aggregate, 0–6 mm (kg/m^3^)	Silica Fume (kg/m^3^)	Plasticizer (kg/m^3^)
HSC	0.30	135	300	522	468	787	150	5.85

**Table 2:** Mechanical properties of CFRP rebars according to catalogue.

**Elastic modulus**	Values in the longitudinal direction of the fibers [13]	
	Mean value	148,000 N/mm^2^
	Minimum value	>140,000 N/mm^2^
**Tensile strength**	Values in longitudinal direction of fibers [13]	
	Mean value	3,100 N/mm^2^
	Minimum value	>2,800 N/mm^2^
	5% Fractile Value	2,900 N/mm^2^
	95% Fractile Value	3,250 N/mm^2^
**Strain at break**	Values in longitudinal direction of fibers [13]	
	Minimum value	>1.70%
**1% elongation**	Minimum tensile force	>110 kN

**Table 3:** Test results

Specimen ^1^	*l*_e_^2^ (mm)	Loading type	ACI		Test		Failure Mode
			*u* ^3^ (MPa)	*f* ^4^ (kN)	*u* (MPa)	*f* (kN)	
S21-25D-T-U	250	C ^5^	6.02	47.31	8.93	70.1	Splitting
S22-25D-M-U	250	M ^6^			11.00	86.4	Splitting Rupture
S23-20D-M-U	200	M	6.71	42.18	12.80	80.4	Splitting
S24-20D-T-U	200	C			11.52	72.4	Pull-out
S25-15D-T-U	150	C	7.86	37.04	13.84	65.2	Pull-out
S26-15D-M-U	150	M			17.74	83.6	Splitting
S27-10D-T-U	100	C	10.15	31.90	11.01	34.6	Pull-out
S28-10D-M-U	100	M			12.32	38.7	Pull-out
S29-5D-M-U	50	M	17.04	26.77	18.02	28.3	Pull-out
S30-5D-T-U	50	C			10.31	16.2	Pull-out

^1^ ribbed 10 mm rebar, ^2^ embedment length, ^3^ average bond stress, ^4^ bond strength, ^5^ cyclic loading, ^6^ monotonic loading.
